# A case of non-immune hydrops fetalis with maternal mirror syndrome diagnosed by trio-based exome sequencing: An autopsy case report and literature review

**DOI:** 10.1016/j.ymgmr.2022.100925

**Published:** 2022-10-14

**Authors:** Sho Tano, Tomomi Kotani, Masato Yoshihara, Noriyuki Nakamura, Seiko Matsuo, Takafumi Ushida, Kenji Imai, Miharu Ito, Yasuyoshi Oka, Emi Sato, Shin Hayashi, Tomoo Ogi, Hiroaki Kajiyama

**Affiliations:** a Department of Obstetrics and Gynecology, Nagoya University Graduate School of Medicine, Nagoya, Aichi, Japan; b Department of Genetics, Research Institute of Environmental Medicine (RIeM), Nagoya University, Nagoya, Aichi, Japan; c Division of Perinatology, Center for Maternal-Neonatal Care, Nagoya University Hospital, Nagoya, Aichi, Japan; d Division of Neonatology, Center for Maternal-Neonatal Care, Nagoya University Hospital, Nagoya, Aichi, Japan; e Department of Human Genetics and Molecular Biology, Graduate School of Medicine, Nagoya University, Nagoya, Aichi, Japan; f Department of Pediatrics and Neonatology, Nagoya City University Graduate School of Medical Sciences, Nagoya, Aichi, Japan; g Department of Genetics, Institute for Developmental Research, Aichi Developmental Disability Center, Kasugai, Aichi, Japan

**Keywords:** exome sequencing, hydrops fetalis, mirror syndrome, Noonan syndrome, *RIT1*

## Abstract

Non-immune hydrops fetalis (NIHF) indicates the risk for stillbirth. Although the causes vary and most NIHFs have no identifiable cause, recent advances in exome sequencing have increased diagnostic rates.

We report a case of NIHF that developed into a giant cystic hygroma complicated by maternal mirror syndrome. Trio-based exome sequencing showed a de novo heterozygous missense variant in the *RIT1* (NM_006912: c.246 T > G [p.F82L]). The *RIT1* variants are known causative variants of Noonan syndrome (NS; OMIM #163950). The location of the *RIT1* variants in the previously reported NS cases with NIHF or/and maternal mirror syndrome was mainly in the switch II region, including the present case.

While a further accumulation of cases is needed, exome sequencing, which can identify the variant type in detail, might help predict the phenotype and severity of NIHF.

## 1 Introduction

Non-immune hydrops fetalis (NIHF) affects approximately 1 in 2000–3000 pregnancies and is a high risk for preterm birth and neonatal complications or death [1]. Standard genetic testing using G-banded karyotyping or chromosomal microarray analysis detects the cause of only 25% of fetuses with NIHF [1]. Therefore, the cause of most fetal NIHF remains unknown. Not only for neonatal complications but also severe NIHF is known to be associated with mirror syndrome, a rare condition characterized by the combination of placental edema, hydrops fetalis, and maternal critical edema [[2], [3], [4]]. It is reported that maternal morbidity increases and fetal mortality are as high as 67% in mirror syndrome [4], but the pathogenesis and effective therapy for mirror syndrome remain unknown. The pregnancy should be terminated to resolve the maternal condition if the fetal hydrops is irreversible [3].

Recent advances in exome sequencing technology have improved diagnostic efficiency [5], especially in genetic disorders. Some genetic disorders, including RASopathies, were possible causes of NIHF [1,6]. One of the RASopathies, Noonan syndrome (NS; OMIM #163950), is an autosomal dominant congenital genetic disorder, and its prevalence is estimated to be 1 in 1000–2500 births [7]. It is a clinically and genetically heterogeneous condition characterized by distinctive facial features, cardiac defects, and other comorbidities [[6], [7], [8], [9], [10], [11], [12], [13], [14], [15], [16], [17]]. Prenatally, NS indicates an increased risk for cystic hygroma or hydrops fetalis [7,9,10]. The diversity of phenotypical features is associated with various genotypes [6]. Several genes are associated with NS, and the genetic variants are identified in approximately 60–70% of patients with NS [7,16]. However, genetic variants relevant for severe fetal or maternal conditions in NIHF remained undetermined.

Here, we report a case of severe fetal hydrops and maternal mirror syndrome. The patient was diagnosed with NS and had a de novo *RIT1* missense variant (NM_006912.6: c.246 T > G [p.F82L]; ClinVar ID: 181522) based on trio-based exome sequencing.

## 2 Case report

**Fig. 1:**
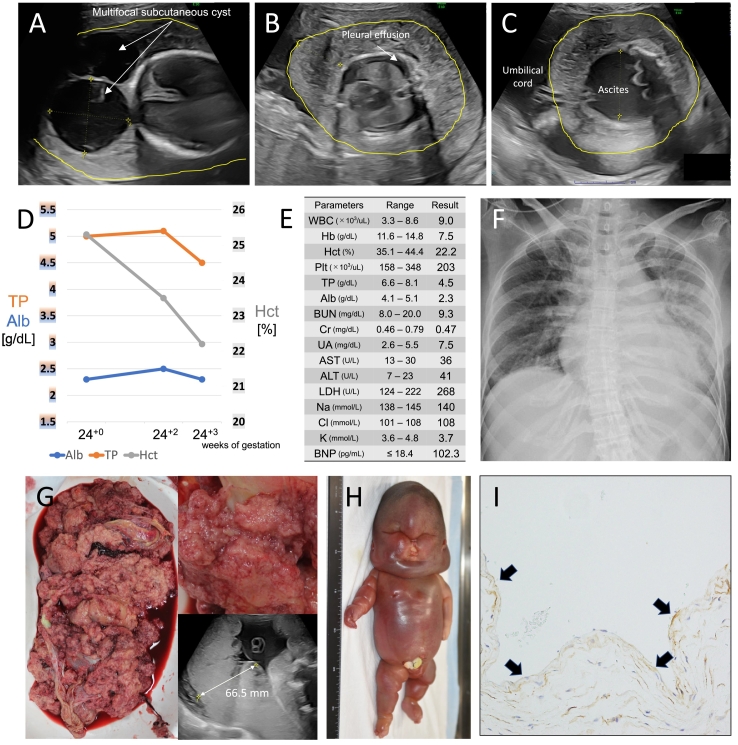
Clinical findings. A-C. Fetal ultrasound findings at 24^+3^ weeks of gestation. The fetus had significant generalized skin edema, cystic hygroma, bilateral pleural effusion, and large amounts of ascites. D. Time course of changes of the maternal blood test. The left axis represents the level of total protein (TP) and albumin (Alb) [mg/dL], and the right axis represents the level of hematocrit (Hct) [%], respectively. E. The results of the maternal blood test at 24^+3^ weeks of gestation. WBC, white blood cell; Hb, hemoglobin; Hct, hematocrit; Plt, platelet count; TP, total protein; Alb, albumin; BUN, blood urea nitrogen; Cr, creatinine; UA, uric acid; AST, aspartate transaminase; ALT, alanine transaminase; LDH, lactate dehydrogenase; Na, sodium; Cl, chloride; K, potassium; BNP, brain natriuretic peptide. F. Maternal chest X-ray at 24^+3^ weeks of gestation. The enlarged cardiothoracic ratio and pulmonary congestion were significant. G. Placenta The left panel shows a macroscopic view of the placenta, showing that the placenta is easily collapsed. The upper right panel is a magnified image of the placenta, showing that the placenta is hydropic. The lower right panel shows the ultrasound finding of thickened placenta with a transverse diameter of 66.5 mm at 24^+2^ weeks of gestation. H. Appearance of newborns. The female neonate had marked edema. I. Pathological findings of the autopsy (×20). Photomicrograph revealed the cyst wall was composed of fibrous connective tissue coated with one layer of the endothelium (D2–40, a lymphatic endothelial marker, arrows).

A 35-year-old G2P0 woman conceived after in vitro fertilization and embryo transfer; her partner was 34 years old. The couple was healthy and non-consanguineous. The singleton fetus presented polycystic hygroma, pleural effusion, and ascites at 16 weeks of gestation. Chromosome karyotype analysis showed a normal female karyotype (46, XX), and a fetal echocardiogram detected no anomaly. The cystic hygroma and hydrops worsened at 24 weeks of gestation, characterized by skin edema, bilateral pleural effusion, and large amounts of ascites (Fig. 1A-C). The placenta was thickened with a transverse diameter of 6.7 cm (Fig. 1G). The mother also had edema and palpitations that gradually worsened after 22 weeks of gestation, while her blood pressure was within the normal range (122/80 mmHg at 24^+3^ weeks of gestation). At 24^+3^ weeks of gestation, hematocrit, total protein, and albumin decreased to 22.2%, 4.5 g/dL, and 2.3 g/dL, respectively (Fig. D-E). She had 1+ proteinuria on dipstick analysis. Chest X-ray was significant for the enlarged cardiothoracic ratio of 58.8% and pulmonary congestion with elevating serum brain natriuretic peptide (BNP) level of up to 102.3 pg/mL (Fig. 1E-F). From these findings, we diagnosed her with mirror syndrome and determined that further continuation of her pregnancy would be dangerous for her. We discussed with her and her partner and decided to perform a Cesarean section at 24^+3^ weeks of gestation due to her volume overload and oxygen demand.

The female newborn was found to have extreme hydrops, and her birth weight and height were 2356 g (>99th percentile) and 31.8 cm (73rd percentile), respectively. The umbilical artery blood pH was 7.40, and 1- and 5-min Apgar scores were 1 and 1, respectively. Neonatal resuscitation was immediately initiated; however, the baby died shortly after birth. The placenta was grossly hydropic and easily collapsed (Fig. 1G). The mother improved steadily after childbirth and was discharged without significant complications.

The autopsy of the newborn showed a large multifocal subcutaneous cyst with a diameter of >10 cm in the posterior neck. Her head and face were deformed by severe skin edema (Fig. 1H). The multifocal subcutaneous cyst contained approximately 70 mL of pale, bloody fluid, and the cyst wall was composed of fibrous connective tissue coated with one layer of endothelium. The endothelium was positive for D2–40 (arrows, Fig. 1I), suggesting fetal cystic lymphangioma. In the skin, dilation of lymphatic vessels was also observed from the dermis to the subcutaneous tissue, suggesting lymphatic congestion. The pleural effusion and ascites were 150 mL in total. Right and left lungs were 6 and 4 g, respectively, suggesting severe pulmonary hypoplasia due to compression of pleural effusion and ascites (17 g on average). The alveolar walls were lined with type II alveolar epithelium, but the air content in the alveolar spaces was poor. No other findings suggest apparent developmental abnormalities or dysfunction, including cardiac defects.

**Fig. 2:**
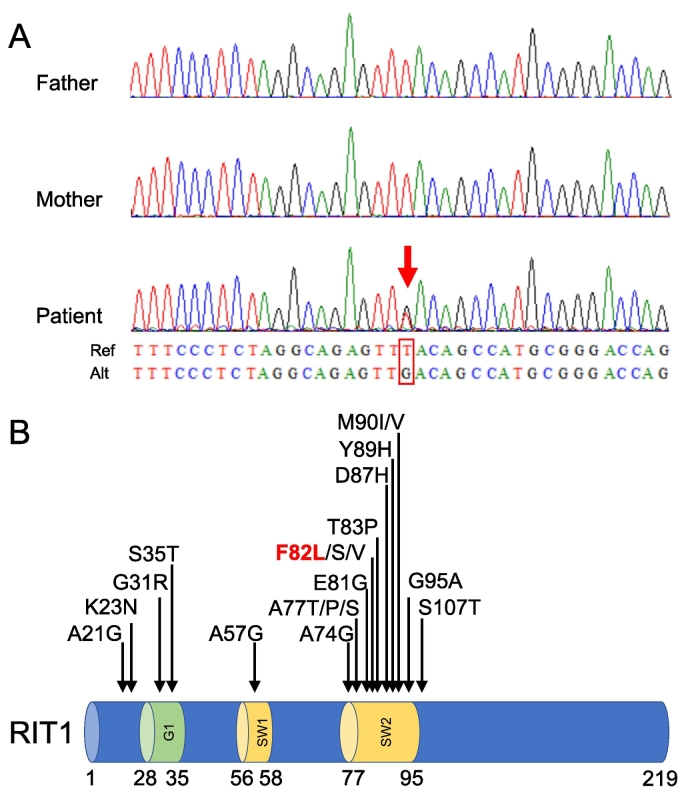
A. Sanger sequencing illustrating a de novo heterozygous *RIT1* missense variant in the present case. B. *RIT1* domain structure (NP_008843.1) and NS-associated amino acid substitutions. Amino acid substitutions identified in a cohort study and case reports (in 12 reports) are given in one-letter code above the structure. The variant in the present case is shown in red. G1, G1 box; SW1, switch I; SW2, switch II.

Trio-based exome sequencing showed a de novo heterozygous missense *RIT1* variant at position 246 (c.246 T > G [p.F82L]) (Fig. 2A). Thus, this patient was diagnosed with Noonan syndrome.

## 3 Discussion

Here, we report a case of severe NS with hydrops and maternal mirror syndrome. According to the autopsy findings, the present neonate died mainly from circulatory failure associated with fetal cystic lymphangioma and fetal hydrops, combined with pleural effusion and respiratory failure due to the immaturity and severe hypoplasia of the lungs. In addition, her mother faced complications due to mirror syndrome. The exome sequence revealed a de novo heterozygous missense variant of *RIT1* (c.246 T > G [p.F82L]), which suggested that NS was the cause of NIHF.

*RIT1* (OMIM # 609591) variants, known causative variants of NS are responsible for approximately 4–9% of all patients with NS [7,9]. *RIT1* has approximately 50% sequence identity to RAS [11] and activates multiple downstream signaling cascades [11,17]. Previously reported *RIT1* variants were all missense variants, and most were gain-of-function variants [7,9,11,17]. The proteins encoded by the mutated *RIT1* gene cause hyperactivation of RAS/MAPK or transactivation of the ELK pathways, participating in the pathogenesis of NS [11]. *RIT1* is expressed ubiquitously in embryonic and adult tissues, and NS patients with *RIT1* variants have a greater incidence of prenatal lymphatic malfunctions, one of the causes of cystic hygroma and hydrops fetalis, and hypertrophic cardiomyopathy (HCM) than in the overall population of patients with NS [6,9].

A total of 20 variants of *RIT1* were identified in patients with NS and were located mainly in the switch II region (SW2) [[6], [7], [8], [9], [10], [11], [12], [13], [14], [15], [16], [17], [18], [19], [20]] (Fig. 2B), corresponding to RAS [[6], [7], [8],^15^]. Table 1 shows the clinical phenotype of *RIT1*-associated NS, including the present and previously reported cases. Cystic hygroma has been detected in 7 patients with *RIT1* variants, including the present case. Three of them had a p.F82L variant, and 2 had a p.M90I or p.M90V variant, which was the close site in the SW2 region. The present case was the second case of mirror syndrome with *RIT1*-associated NS. The previous one had the de novo p.M90V variant, which was also the close site in the SW2 region [20]. Our patient also had a de novo SW2 region variant, suggesting fetal factors might affect the progression to maternal mirror syndrome, while the evidence is limited. It is reported that fetal hydrops or cystic hygroma could affect the placental-derived factor production [21], possibly involved in the pathogenesis of mirror syndrome [22]. Further case accumulation is needed to investigate the association between the RIT1 domain and cystic hygroma or mirror syndrome. Although the pathologic association remains unclear, predicting mirror syndrome in earlier gestation would be very helpful for the mother and her partner.

**Table 1:** Clinical phenotype of *RIT1*-associated NS.

RIT1 Mutation		Ref No.	Age	Sex	Inheritance	Mirror Syndrome	Prenatal findings			Cardiac anomalies			
Region	Position						CH	NE	Others	HCM	ASD	VSD	Others
	p.K23N	13	10m	M	ND	−	−	−	PH	−	−	−	PS

SW1	p.G31R	10	8y	F	Inherited	ND	−	−	−	−	−	−	PS
		10	47y	F	Not known	ND	ND	ND	ND	−	−	−	PS

	p.S35T	10	1y	F	De novo	ND	−	−	−	−	−	−	PS
		10	5y	F	De novo	ND	−	−	PH	**+**	−	−	PS
		10	11y	M	Not known	ND	−	−	−	**+**	−	−	PS
		6	1y	F	ND	ND	−	**+**	−	−	**+**	−	PS
		6	3y	M	ND	ND	−	**+**	−	**+**	−	−	PS, AS
		8	2y	M	ND	ND	−	−	−	−	−	−	PS
		19	3m	ND	De novo	ND	−	−	PH	**+**	−	−	PS
15		FD	ND	ND	−	**+**	**+**	PE	ND	ND	ND	ND

SW2	p.A57G	10	11y	F	Not known	ND	−	−	−	**+**	−	−	PS
		10	1y	F	De novo	ND	−	**+**	PE, PH	**+**	−	−	PS
		10	6y	M	Inherited	ND	−	−	PH	−	**+**	**+**	PS
		10	29y	F	Not known	ND	ND	ND	ND	−	−	−	PS
		10	2m	M	Not known	ND	−	**+**	PH	**+**	−	**+**	PS
		10	6m	F	Not known	ND	−	−	−	**+**	**+**	**+**	PS
		10	7y	M	De novo	ND	−	**+**	PE, PH	**+**	−	−	MVA
		10	5m	M	Inherited	ND	−	−	PH	**+**	−	−	PS
		10	32y	F	Not known	ND	ND	ND	ND	−	−	−	PS
		6	15d	F	ND	ND	−	**+**	PE, PH	**+**	−	−	PDA
		6	5y	F	ND	ND	−	**+**	PH	−	−	−	PS, PDA
		6	11 m	F	ND	ND	−	−	PE, PH	−	**+**	**+**	PS, PDA
		8	27y	F	ND	ND	ND	ND	ND	**+**	−	−	PS
		8	28y	M	ND	ND	−	−	−	−	−	−	PS
		12	2y	F	ND	ND	ND	ND	ND	−	−	−	PS
		11	15y	F	ND	−	**+**	**+**	−	**+**	**+**	**+**	PS
		18	ND	M	ND	ND	ND	ND	ND	**+**	−	−	PS

	p.A77T	10	3y	F	Not known	ND	−	−	PH	−	**+**	−	PS
		10	21y	M	Not known	ND	ND	ND	ND	−	−	−	PS, MVA
		6	4y	F	ND	ND	−	−	PE	−	**+**	**+**	
		6	1y	F	ND	ND	−	−	PH	**+**	**+**	−	PS
		19	ND	M	ND	ND	ND	ND	ND	−	−	**+**	PS

	p.A77P	12	22y	F	ND	ND	ND	ND	ND	−	**+**	−	PS

	p.A77S	6	3y	F	ND	ND	−	−	PE, PH	**+**	**+**	−	

	p.E81G	9	ND	ND	ND	ND	ND	ND	ND	−	−	**+**	PS
		14	14y	M	ND	−	−	−	−	**+**	−	−	

	p.F82V	10	13y	F	De novo	ND	−	**+**	PE, FH	**+**	−	−	PS
		12	6y	F	ND	ND	−	**+**	−	−	**+**	**+**	PS, PDA,
		7	10y	F	ND	ND	ND	ND	ND	−	−	−	PS, PDA

	p.F82S	10	29y	M	Not known	ND	−	−	−	−	−	−	−

	**p.F82L**	**Present case**	**0d**	**F**	**De novo**	**+**	**+**	**+**	**PE**	**−**	**−**	**−**	**−**
		10	5y	F	De novo	ND	−	**+**	PE	**+**	−	−	MVA
		10	38y	F	Not known	ND	ND	ND	ND	−	−	−	PS, MVA
		10	5y	F	Not known	ND	−	−	PH	**+**	−	−	PS
		10	11y	M	Not known	ND	−	−	PH	**+**	**+**	−	PS
		10	13y	M	Not known	ND	−	−	−	−	−	−	PS
		10	1y	F	Not known	ND	−	**+**	PE	**+**	**+**	−	PDT
		6	4y	M	ND	ND	−	**+**	PE	**+**	−	−	−
		6	4y	M	ND	ND	−	−	PE	**+**	**+**	−	−
		6	8d	M	ND	ND	**+**	**+**	−	ND	ND	ND	ND
		18	10d	ND	De novo	ND	−	**+**	−	**+**	−	−	PS
		8	19y	M	ND	ND	**+**	**+**	−	−	**+**	**+**	PS

	p.T83P	10	16y	F	Not known	ND	−	−	−	**+**	−	−	−

	p.D87H	18	ND	M	Not known	ND	−	−	−	**+**	**+**	−	PS

	p.Y89H	9	ND	ND	Not known	ND	ND	ND	ND	**+**	−	−	PS

	p.M90I	10	3y	F	Not known	ND	−	−	−	−	−	−	PS, MVA
		6	5y	M	ND	ND	−	−	PH	−	−	−	PS
		7	5y	F	ND	ND	**+**	**+**	**−**	**+**	−	−	PS
		11	2y	M	ND	**−**	−	−	PH	**+**	**+**	**+**	PS, PDA

	p.M90V	20	FD	M	De novo	**+**	ND	ND	FH	ND	ND	ND	ND
		15	FD	ND	ND	−	**+**	**+**	PE	ND	ND	ND	ND

	p.G95A	10	8y	M	Inherited	ND	−	−	−	−	−	−	PS
		10	42y	F	Not known	ND	−	−	−	−	**+**	**+**	−
10		14y	F	Inherited	ND	−	−	−	−	−	−	PS
10		24y	M	Not known	ND	−	−	−	−	−	−	MVA
10		15y	F	Not known	ND	−	**+**	PH	−	**+**	−	−
10		11y	M	Not known	ND	−	**+**	−	−	**+**	−	PS
6		15y	M	ND	ND	−	−	PH	−	−	−	PS
6		3mo	F	ND	ND	−	−	PE	−	−	−	PDA
8		16y	F	ND	ND	−	−	PH	−	**+**	−	PS, PDA
8		27y	M	ND	ND	−	−	−	**+**	−	−	−
12		46y	F	ND	ND	ND	ND	ND	−	−	−	PS
12		18y	M	ND	ND	ND	ND	ND	−	−	−	PS
7		17y	F	ND	ND	ND	ND	ND	−	−	**+**	PS
7		13y	F	ND	ND	ND	ND	ND	−	**+**	−	PS
16		11y	F	De novo	ND	ND	ND	ND	−	−	−	PS

Bold letters indicate the present case. ND, no data; FD, intrauterine fetal demise; PH, polyhydramniosis; CH, cystic hygroma; NE, fetal nuchal edema; PE, fetal preural effusion; FH, fetal hydrops; PS, pulmonary stenosis; HCM, hypertrophic cardiomyopathy; ASD, atrial septal defect; AS, aortial stenosis; VSD, ventricula septal defect; PDA, patent ductus arteriosus; MVA, mytral valve anomalies; LE, leg edema; CT cataract; ASC, ascites.

In conclusion, exome sequencing was valuable in NIHF, where chromosome testing could not identify the cause. The present and previous cases speculated that exome sequencing, which can identify the variant type in detail, might help predict the phenotype and severity of NIHF and its association with mirror syndrome. However, further accumulation of cases is needed to confirm this speculation.

## Funding

This work was supported by the special coordination funds for Rare and Intractable Diseases Project of Japan from the 10.13039/100009619Japan Agency for Medical Research and Development (T.O.).

## Author contributions

Conceptualization and planning: Sho Tano, Tomomi Kotani, and Tomoo Ogi; clinical data collection and interpretation: Masato Yoshihara, Seiko Matsuo, Takafumi Ushida, Kenji Imai, and Miharu Ito; bioinformatics analysis: Noriyuki Nakamura, Yasuyoshi Oka, Emi Sato, and Shin Hayashi; supervision: Tomoo Ogi and Hiroaki Kajiyama.

All authors discussed, read, and approved the manuscript in its final form.

## Ethics statement

This study was approved by the Ethical Committee for the Study of Human Gene Analysis at Nagoya University Graduate School of Medicine (approval number: 2018–0288). The patient's parents gave written consent for the publication of the clinical information, including photographs.

## Declaration of Competing Interest

The authors have no conflicts of interest to declare.

## Data Availability

Data will be made available on request.

## References

[1] Sileo F.G. (2020). Non-immune fetal hydrops: etiology and outcome according to gestational age at diagnosis. Ultrasound Obstet. Gynecol..

[2] Graham N. (2012). Placental expression of anti-angiogenic proteins in mirror syndrome: a case report. Placenta.

[3] Gedikbasi A. (2011). Preeclampsia due to fetal non-immune hydrops: mirror syndrome and review of literature. Hypertens Pregnancy.

[4] Allarakia S. (2017). Characteristics and management of mirror syndrome: a systematic review (1956-2016). J. Perinat. Med..

[5] Petrovski S. (2019). Whole-exome sequencing in the evaluation of fetal structural anomalies: a prospective cohort study. Lancet.

[6] Yaoita M. (2016). Spectrum of mutations and genotype-phenotype analysis in Noonan syndrome patients with RIT1 mutations. Hum. Genet..

[7] Gos M. (2014). Contribution of RIT1 mutations to the pathogenesis of Noonan syndrome: four new cases and further evidence of heterogeneity. Am. J. Med. Genet. A.

[8] Bertola D.R. (2014). Further evidence of the importance of RIT1 in Noonan syndrome. Am. J. Med. Genet. A.

[9] Aoki Y. (2013). Gain-of-function mutations in RIT1 cause Noonan syndrome, a RAS/MAPK pathway syndrome. Am. J. Hum. Genet..

[10] Kouz K. (2016). Genotype and phenotype in patients with Noonan syndrome and a RIT1 mutation. Genet Med..

[11] Koenighofer M. (2016). Mutations in RIT1 cause Noonan syndrome - additional functional evidence and expanding the clinical phenotype. Clin. Genet..

[12] Chen P.C. (2014). Next-generation sequencing identifies rare variants associated with Noonan syndrome. Proc. Natl. Acad. Sci. U. S. A..

[13] Nemcikova M. (2016). A novel heterozygous RIT1 mutation in a patient with Noonan syndrome, leukopenia, and transient myeloproliferation-a review of the literature. Eur. J. Pediatr..

[14] Ramond F. (2017). Expanding the cardiac spectrum of Noonan syndrome with RIT1 variant: left main coronary artery atresia causing sudden death. Eur. J. Med. Genet..

[15] Stevens B. (2017). Response to: Milosavljevic et al. “two cases of RIT1 associated Noonan syndrome: further delineation of the clinical phenotype and review of the literature”. Am. J. Med. Genet. A.

[16] Koh A.L. (2019). The spectrum of genetic variants and phenotypic features of southeast Asian patients with Noonan syndrome. Mol. Genet Genomic. Med..

[17] Li X. (2019). Molecular and phenotypic spectrum of Noonan syndrome in Chinese patients. Clin. Genet..

[18] Leung G.K.C. (2018). Integrating functional analysis in the next-generation sequencing diagnostic pipeline of RASopathies. Sci. Rep..

[19] Andelfinger G. (2019). Hypertrophic cardiomyopathy in Noonan syndrome treated by mek-inhibition. J. Am. Coll. Cardiol..

[20] Milosavljevic D. (2016). Two cases of RIT1 associated Noonan syndrome: further delineation of the clinical phenotype and review of the literature. Am. J. Med. Genet. A.

[21] Alvarez-Nava F. (2015). Elevated second-trimester maternal serum beta-human chorionic gonadotropin and amniotic fluid alpha-fetoprotein as indicators of adverse obstetric outcomes in fetal turner syndrome. J. Obstet. Gynaecol. Res..

[22] Hirata G. (2016). Clinical characteristics of mirror syndrome: a comparison of 10 cases of mirror syndrome with non-mirror syndrome fetal hydrops cases. J. Matern. Fetal Neonatal Med..

